# Marker and readout genes for defense priming in *Pseudomonas cannabina* pv. alisalensis interaction aid understanding systemic immunity in Arabidopsis

**DOI:** 10.1038/s41598-024-53982-5

**Published:** 2024-02-12

**Authors:** Andrea J. Sistenich, Lisa Fürtauer, Franziska Scheele, Uwe Conrath

**Affiliations:** 1 Plant Biochemistry and Molecular Biology Group, Department of Plant Physiology, RWTH Aachen University, 52062 Aachen, Germany; 2 Plant Molecular Systems Biology Group, Department of Plant Physiology, RWTH Aachen University, 52062 Aachen, Germany

**Keywords:** Plant immunity, Plant signalling, Plant stress responses

## Abstract

Following localized infection, the entire plant foliage becomes primed for enhanced defense. However, specific genes induced during defense priming (priming-marker genes) and those showing increased expression in defense-primed plants upon rechallenge (priming-readout genes) remain largely unknown. In our *Arabidopsis thaliana* study, genes *AT1G76960* (function unknown), *CAX3* (encoding a vacuolar Ca^2+^/H^+^ antiporter), and *CRK4* (encoding a cysteine-rich receptor-like protein kinase) were strongly expressed during *Pseudomonas cannabina* pv. alisalensis-induced defense priming, uniquely marking the primed state for enhanced defense. Conversely, *PR1* (encoding a pathogenesis-related protein), *RLP23* and *RLP41* (both encoding receptor-like proteins) were similarly activated in defense-primed plants before and after rechallenge, suggesting they are additional marker genes for defense priming. In contrast, *CASPL4D1* (encoding Casparian strip domain-like protein 4D1), *FRK1* (encoding flg22-induced receptor-like kinase), and *AT3G28510* (encoding a P loop-containing nucleoside triphosphate hydrolases superfamily protein) showed minimal activation in uninfected, defense-primed, or rechallenged plants, but intensified in defense-primed plants after rechallenge. Notably, mutation in only priming-readout gene *NHL25* (encoding NDR1/HIN1-like protein 25) impaired both defense priming and systemic acquired resistance, highlighting its previously undiscovered pivotal role in systemic plant immunity.

## Introduction

After a localized leaf infection by necrogenic microbes, the entire plant foliage can become primed for enhanced activation of defense upon rechallenge (this immunological condition is subsequently referred to in this paper as priming)^1–3^. Primed leaves respond more strongly to reinfection by different pathogens or physical injury and they frequently express resistance to multiple diseases^1–5^. One such priming-caused broad-spectrum disease resistance response is systemic acquired resistance (SAR), which wards off biotroph and hemibiotroph pathogens^1–5^. Different from the full activation of defense responses upon initial infection, priming causes only low fitness costs^6,7^. In addition, priming is hardly prone to pathogen adaptation^3,7^. Therefore, exploiting priming is promising for practical agronomic use and interesting as a paradigm for plant signal transduction as well^3,8^.

Priming comprises enhanced levels in the plasma membrane of microbial pattern receptors and coreceptors^9^, such as protein kinase flagellin-sensing 2 (FLS2, recognizing the bacterial flagellin epitope flg22), brassinosteroid-insensitive 1-associated receptor kinase 1 (BAK1, a coreceptor of FLS2), and chitin elicitor receptor kinase 1 (CERK1, the chitin and peptidoglycan receptor or coreceptor, respectively)^9^. The enhanced presence of microbial pattern receptors and coreceptors in the plasma membrane of primed cells increases the responsiveness to microbes harboring flagellin, chitin, or peptidoglycan^9^. Consistent with the role of FLS2, BAK1, and CERK1 in activating mitogen-activated protein kinase (MPK) signaling relays^10,11^, priming likewise encompasses enhanced levels of dormant, but activable MPK3 and MPK6 molecules^12^. Because of the enhanced levels of MPK3 and MPK6 in primed cells, more of these enzymes are activated upon stimulation of the microbial-pattern receptors thus amplifying the transducing signal and, ultimately, leading to enhanced defense^12^.

In addition to the enhanced levels of microbial pattern receptors and activatable MPK3 and MPK6 molecules, priming includes covalent modification of DNA and histones in the promoter of defense genes, such as those encoding WRKY transcription factors with a role in plant defense^13,14^. The modification of DNA and histones primes the affected gene for enhanced transcription after further stimulation^13–15^. Together, the enhanced levels of microbial-pattern receptors and dormant MPKs as well as the mounting of gene-conditioning chromatin modifications provide a memory to the priming-inducing event in that they prime cells for the superinduction of defense responses by physical rechallenge or microbial reinfection, associated with development of stress tolerance and SAR^3^.

Surprisingly, although priming received much attention both as a promising concept for plant protection^1–3,5,8^ and a paradigm for cellular signal transduction^1–3,12,13,15^, the identity of genes that are specifically expressed during priming (referred to here as priming-marker genes) or whose expression is stronger in primed than unprimed plants after rechallenge (referred to as priming-readout genes) remain largely unknown. This is particularly surprising for the intensively studied interaction of *Arabidopsis thaliana* (Arabidopsis) with *Pseudomonas cannabina* pv. alisalensis (Pcal; formerly called *Pseudomonas syringae* pv. maculicola ES4326)^16–18^. Knowing the identity of marker and readout genes for priming would equip the plant research community with novel tools for the research into priming, help expanding the knowledge of the phenomenon, and support its translation to agricultural practice, e.g., through identifying priming-inducing chemistry or by breeding for enhanced sensitivity to be primed^8^.

So far, we and others often used *WRKY6*, *WRKY29*, and *WRKY53* as readout genes to assess priming in Arabidopsis^13,14^. Monitoring their expression advanced the research into priming but the weight of these loci as priming-readout genes remains unclear. A genome-wide record of marker and readout genes for priming simply was missing. We recently used formaldehyde-assisted isolation of regulatory DNA elements (FAIRE) to provide a genome-wide map of regulatory DNA sites in the primed foliage of Arabidopsis plants with local Pcal infection^19^. Supplemental whole-transcriptome shotgun sequencing of mRNA transcripts from systemic leaves of primed and unprimed plants, both before and after physical rechallenge, disclosed all Arabidopsis genes with expression before (possible priming-marker genes) and enhanced expression after (possible priming-readout genes) rechallenge. So far, these datasets remain insufficiently explored and marker genes for individual immunological conditions (primed or unprimed both before and after rechallenge) unconfirmed. Here, we introduce genes that we validated as suitable marker or readout genes for priming in the interaction of Arabidopsis with Pcal. Based on *in-silico* analyses we also predict interaction networks and subcellular mapping of the proteins encoded by marker and readout genes for priming. We also demonstrate that mutation of solely priming-readout gene *NHL25* (encoding NDR1/HIN1-like protein 25) attenuates Pcal-induced priming for enhanced defense gene activation and impairs SAR.

## Results

### Spotting and validating marker genes for priming

**Figure 1:**
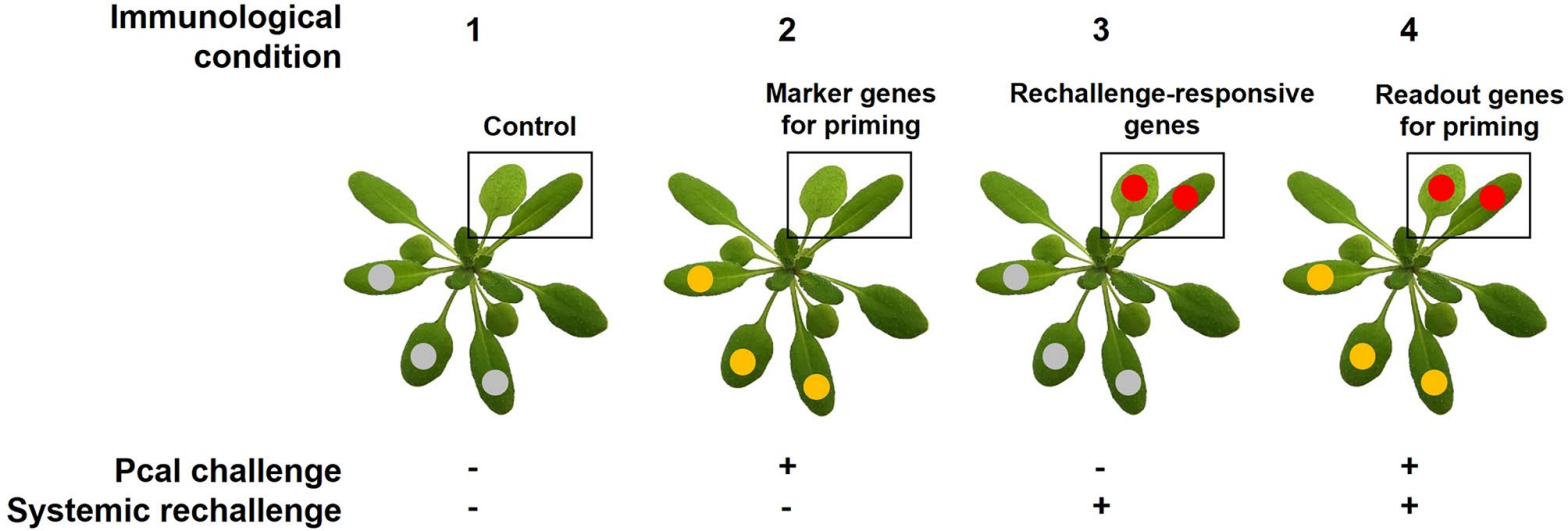
Immunological conditions used to identify marker genes for priming, rechallenge-responsive genes and readout genes for priming in systemic leaves of Arabidopsis plants. Unfilled boxes refer to leaves that were harvested and analyzed for gene expression. Grey circles indicate mock treatment, yellow circles indicate local Pcal infection, and red circles indicate systemic rechallenge. Condition 1 represents plants with local mock infection without any systemic treatment. They served as a control for the other treatments.

In Arabidopsis, priming and SAR exhibit strong activation 3–4 days after Pcal infection but subsequently decline^19^. To identify and validate potential marker and readout genes associated with priming in Arabidopsis, we reevaluated Supplementary Dataset S1 from our previous publication by Baum et al.^19^. This dataset comprises genes whose expression is either activated or repressed in one or more of four immunological conditions as depicted in Fig. 1. These conditions include mock challenge on local leaves (condition 1, control) and Pcal challenge on local leaves (condition 2, systemically primed), both before (conditions 1 and 2) and after systemic rechallenge (conditions 3 and 4). Additionally, the dataset, that originates from systemic leaves at the 3rd day after mock or Pcal challenge, provides information regarding changes in chromatin accessibility in the promoter of individual genes across the four immunological conditions, as determined by FAIRE^19,20^.

Of Supplementary Dataset S1^19^ we first used the data subset of systemic leaves in immunological condition 2 (referred to as data subset ncP in^19^) which should contain genes whose expression is activated during priming. We compared it with the data subset of systemic leaves in condition 4 (referred to as data subset pC in^19^) to spot those genes whose activated expression in primed leaves is reduced again after rechallenge (named pC data subset in^19^). Genes with both activated expression in condition 2 and repression in condition 4 are most promising to be exclusive marker genes for priming. This is particularly true if they also have priming-associated open chromatin in the promoter, as inferred from the FAIRE data subset of Baum et al.^19^.

**Figure 2:**
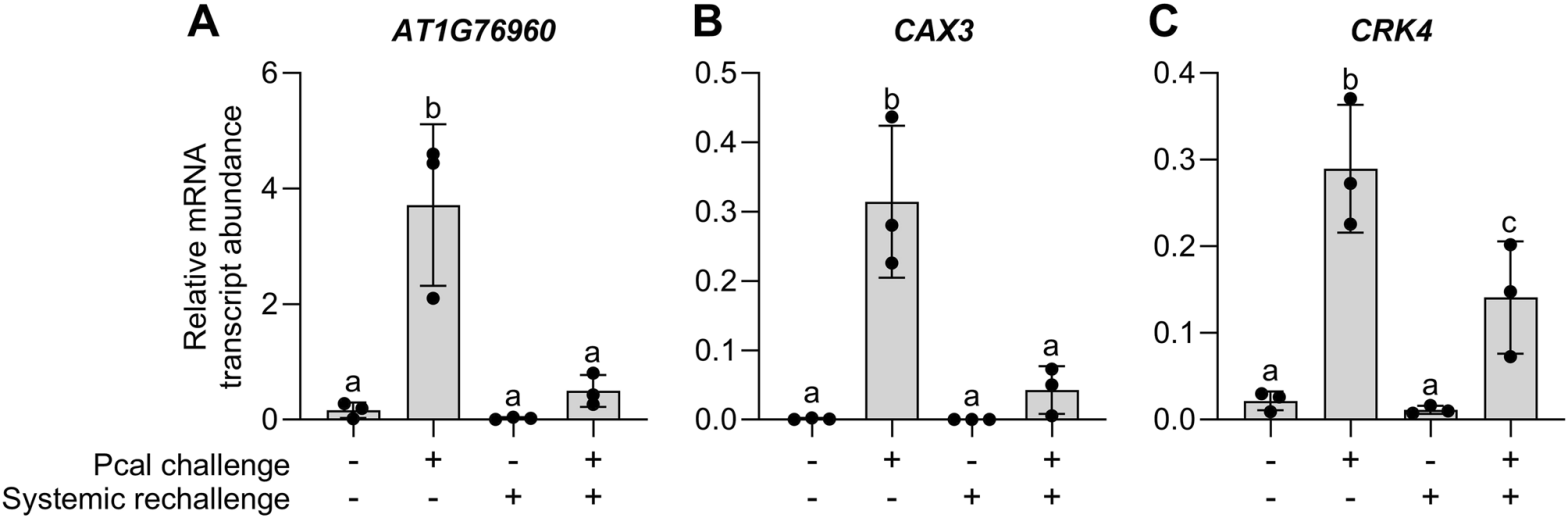
Expression of *AT1G76960* (**A**), *CAX3* (**B**), and *CRK4* (**C**) is particularly activated during priming. Five-week-old Arabidopsis plants were infiltrated on three leaves with MgCl_2_ (mock inoculation;  − Pcal) or a Pcal suspension in MgCl_2_ (+ Pcal). Three days later, untreated leaves of both sets of plant were left untreated (− systemic rechallenge) or rechallenged by the infiltration of water (+ systemic rechallenge). Three hours later, the systemic leaves were harvested and analyzed for the expression of specified genes. Relative mRNA transcript abundance was determined by RT-qPCR and normalized to the expression of *ACTIN2*. Shown are the mean values and SD of three independent experiments, each with two plants. Statistical significance was determined using Ordinary one-way ANOVA. (**A**; **B**), *P* < 0.001; (**C**), *P* < 0.05.

We used Microsoft Excel to identify the top ten genes expressed in primed leaves (condition 2), exhibiting high FAIRE values, and being repressed upon systemic rechallenge (condition 4) (i.e., low pC values in Supplementary Dataset S1 in^19^), as shown in Table 1. Additionally, we conducted in-silico identification of the top condition 4 versus condition 3 (influence of priming on systemic rechallenge) genes with high FAIRE values, also listed in Table 1. Subsequently, in the lab we reevaluated the expression of these genes in each of the four immunological conditions. Among the analyzed genes, *AT1G76960* (encoding a protein with unknown function) (Fig. 2A), *CAX3* (a vacuolar Ca^2+^/H^+^ antiporter) (Fig. 2B), and *CRK4* (a cysteine-rich receptor-like protein kinase) (Fig. 2C) exhibit notable expression during Pcal-induced priming in infection-free systemic leaves (condition 2). In contrast, their expression is not, or to a lesser extent, observed in control (condition 1) or rechallenged leaves (condition 3) or in rechallenged leaves after priming (condition 4) (Fig. 2). The exclusive expression of these genes signifies the primed state characterized by enhanced defense readiness. Another marker gene for priming, *AT5G64190* (encoding a neuronal PAS-domain protein with an unknown function), also demonstrates expression, albeit with lower overall levels compared to the previously mentioned genes (Supplementary Fig. S1).

**Table 1:** Top candidates for marker and readout genes for priming.

Gene locus	Gene symbol	Gene description	ncP logFC	pC logFC	cP logFC	FAIRE logRatio
Top exclusive marker genes for priming (expressed during priming and repressed after rechallenge in primed leaves (immunological condition 2 compared to condition 4; Fig. 1)						
*AT5G55440*	*ATDOA16*	DUF295 ORGANELLAR A 16, F− box protein	7.08	− 1.85	5.16	2.98
*AT5G22380*	*NAC090*	NAC domain containing protein 90	6.01	− 1.19	1.54	3.47
*AT3G25010*	*RLP41*	Receptor− like protein 41	5.93	− 0.15	4.53	5.06
*AT3G60470*		Transmembrane protein, putative DUF247	5.75	− 0.61	3.01	3.01
*AT5G64190*		Neuronal PAS domain protein	5.72	− 1.77	1.11	2.88
*AT2G37820*		Cysteine/histidine− rich C1 domain family protein	5.19	− 0.04	1.75	4.55
*AT2G32680*	*RLP23*	NLP20 LRR receptor protein involved in PAMP− mediated immunity	4.62	− 0.10	2.84	4.14
*AT5G47850*	*CCR4*	Crinkly 4− related 4	4.44	− 0.38	2.52	4.34
*AT3G45860*	*CRK4*	Cysteine− rich receptor− like protein kinase CRK 4	4.44	− 0.28	2.84	4.36
*AT1G76960*		Unknown protein, contains WRKY40 binding motifs	4.24	− 0.90	2.79	2.02
Top priming− responsive genes (expressed during priming and unaffected after further rechallenge in primed leaves) (condition 2 compared to condition 4)						
*AT3G45330*	*LECRK− I.1*	Concanavalin A− like lectin protein kinase family protein	8.63	4.14	5.88	6.99
*AT4G10860*		Hypothetical protein	7.98	2.70	8.60	2.40
*AT2G26400*	*ARD3*	Acireductone dioxygenase 3	7.94	1.64	5.40	2.06
*AT2G14610*	*PR1*	Pathogenesis− related gene 1	7.51	2.34	7.17	6.24
*AT3G44326*	*CFB*	Cytokinin induced F− Box protein	7.43	1.49	3.16	3.02
*AT4G37010*	*CEN2*	Centrin 2	7.36	2.33	5.14	3.71
*AT1G71390*	*RLP11*	Receptor− like protein 11	7.31	3.21	5.09	4.52
*AT3G46080*	*ZAT8*	C2H2− type zinc finger family protein	7.26	2.21	2.50	3.03
*AT5G62480*	*GSTU9*	Glutathione S− transferase tau 9	7.12	4.63	3.13	2.32
*AT5G55440*	*ATDOA16*	DUF295 ORGANELLAR A 16, F− box protein	7.08	− 1.85	5.16	2.98
Top genes expressed because of priming in systemic rechallenge condition (condition 3 compared to condition 4)						
*AT5G24540*	*BGLU31*	Beta glucosidase 31	2.63	4.18	10.10	4.22
*AT4G10860*		Hypothetical protein	7.98	2.70	8.60	2.40
*AT4G05540*		P loop− containing nucleoside triphosphate hydrolases superfamily protein	7.02	1.47	8.42	2.58
*AT2G25440*	*RLP20*	Receptor− like protein 20	4.96	2.34	7.24	4.73
*AT2G14610*	*PR1*	Pathogenesis− related gene 1	7.51	2.34	7.17	6.24
*AT3G44350*	*NAC061*	NAC domain− containing protein 61	3.34	2.99	6.82	4.83
*AT1G66870*		Carbohydrate− binding X8 domain superfamily protein	0.00	7.04	6.78	4.78
*AT3G51860*	*CAX3*	Vacuolar Ca^2+^/H^+^ antiporter	5.83	0.15	6.64	3.32
*AT5G39390*		Leucine− rich repeat protein kinase family protein	3.26	3.30	6.48	4.55
*AT1G13520*		Hypothetical protein DUF1262	6.26	4.55	6.39	2.92
*AT3G28230*	*MED20A*	Component of mediator complex	3.34	2.90	6.15	3.29
*AT3G28510*		P loop− containing nucleoside triphosphate hydrolases superfamily protein	5.97	3.56	6.01	3.67
Top genes expressed after rechallenge in primed leaves (condition 4)						
*AT3G45060*	*NRT2.6*	High− affinity nitrate transporter 2.6	− 0.38	9.82	2.34	2.34
*AT2G39530*	*CASPL4D1*	Uncharacterized protein family UPF0497	0.49	9.02	2.68	5.64
*AT1G65481*		Transmembrane protein	0.39	8.79	2.92	3.80
*AT2G36690*	*GIM2*	2− oxoglutarate and Fe(II)− dependent oxygenase superfamily protein	− 1.81	8.74	3.37	2.45
*AT4G12500*		Bifunctional inhibitor/lipid− transfer protein/seed storage 2S albumin superfamily protein	2.02	8.09	2.29	2.33
*AT5G36970*	*NHL25*	NDR1/HIN1− like 25	− 4.50	8.08	4.04	2.35
*AT2G45220*	*PME17*	Pectin methylesterase involved in pectin remodeling	2.49	7.60	3.30	2.55
*AT2G19190*	*FRK1*	Flg22− induced receptor− like kinase 1	2.38	7.56	2.91	3.95
*AT4G23550*	*WRKY29*	WRKY DNA− binding protein 29	0.92	7.26	1.35	3.67
*AT1G69930*	*GSTU11*	Glutathione S− transferase TAU 11	− 0.19	7.13	3.41	3.08

ncP, genes with highest ncP logFC and FAIRE logRatio > 2; ncP up – pC down, genes with highest ncP logFC, negative pC logFC and a FAIRE logRatio > 2; cP, genes with highest cP logFC and a FAIRE logRatio > 2; pC, genes with highest pC logFC and a FAIRE logRatio > 2. ncP, genes expressed during priming; pC, genes expressed after challenge of primed leaves; cP, genes expressed because of priming in systemic challenge condition. logFC, logFold change; FAIRE, formaldehyde-assisted isolation of regulatory DNA elements. Table based on Supplementary Dataset S1 of Baum et al. ^19^ with gene symbols and descriptions being updated according to TAIR and Araport11.

**Figure 3:**
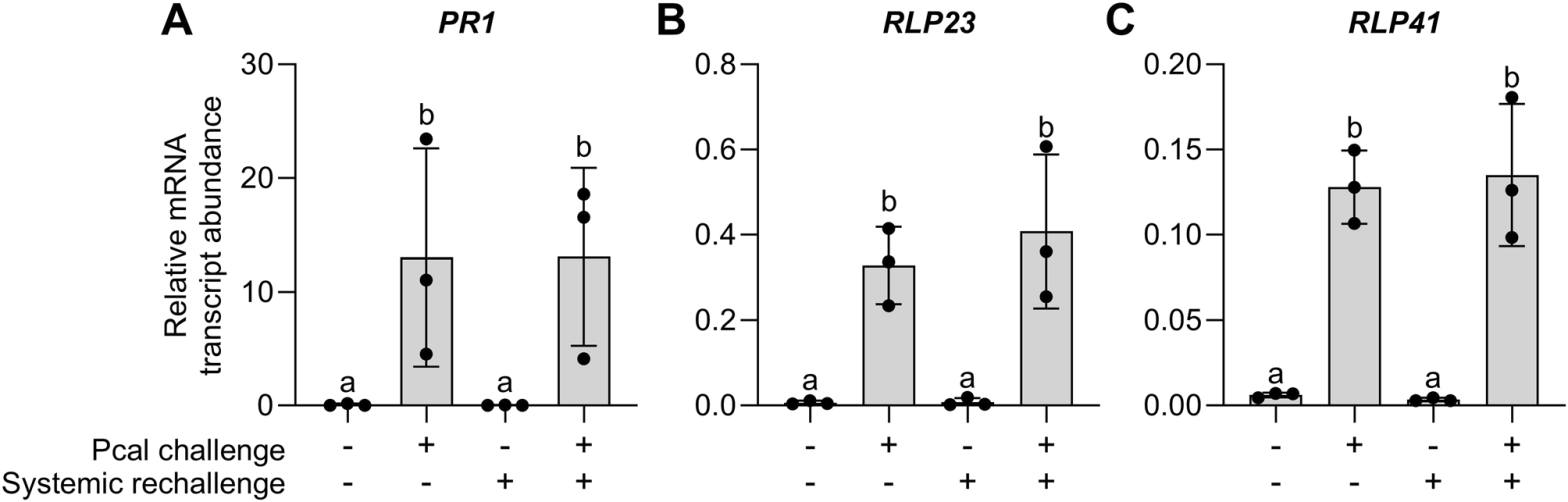
Expression of *PR1* (**A**), *RLP23* (**B**), and *RLP41* (**C**) is associated with priming. Experimental setup, data and statistical analyses were performed as in Fig. 2. (**A**), *P* < 0.05; (**B**), *P* < 0.01; (**C**), *P* < 0.001.

In contrast to the previously mentioned genes, *PR1* (encoding a pathogenesis-related protein) (Fig. 3A), *RLP23* (encoding a receptor-like protein) (Fig. 3B), and *RLP41* (encoding another receptor-like protein) (Fig. 3C) show similar activation levels in primed leaves before (condition 2) and after rechallenge (condition 4) (Fig. 3). These genes can also serve as marker genes for priming, as their expression is activated in the primed state and remains essentially unchanged upon rechallenge (Fig. 3). *CCR4* (crinkly 4-related 4), *AT4G05540* (encoding a P loop-containing nucleoside triphosphate hydrolase superfamily protein), and *AT1G66870* (encoding a carbohydrate-binding X8-domain superfamily protein) also belong to this group of priming-marker genes, albeit with lower overall expression (Supplementary Fig. S2A–C).

### Spotting and validating readout genes for priming

To identify potential readout genes for priming in Arabidopsis, we conducted an *in-silico* reevaluation of our gene-expression data subsets of plants in condition 4 (primed and later rechallenged; pC data subset in^19^), condition 3 (influence of priming on systemic rechallenge; cP subset in^19^), condition 2 (no rechallenge but priming; ncP subset in^19^), and FAIRE (Table 1; details in^19^). We in-silico identified eight genes that exhibited high expression in immunological condition 4 and possessed a chromatin accessibility (FAIRE) value of > 2^19^. In our analysis, we also included previously used priming-readout genes, *WRKY6* (ranked #84 in the pC gene list in^19^), and *WRKY53* (ranked #232 in their pC gene list; see Supplementary Table S1,^19^), to assess their weight as readout genes for priming^13,14^.

**Figure 4:**
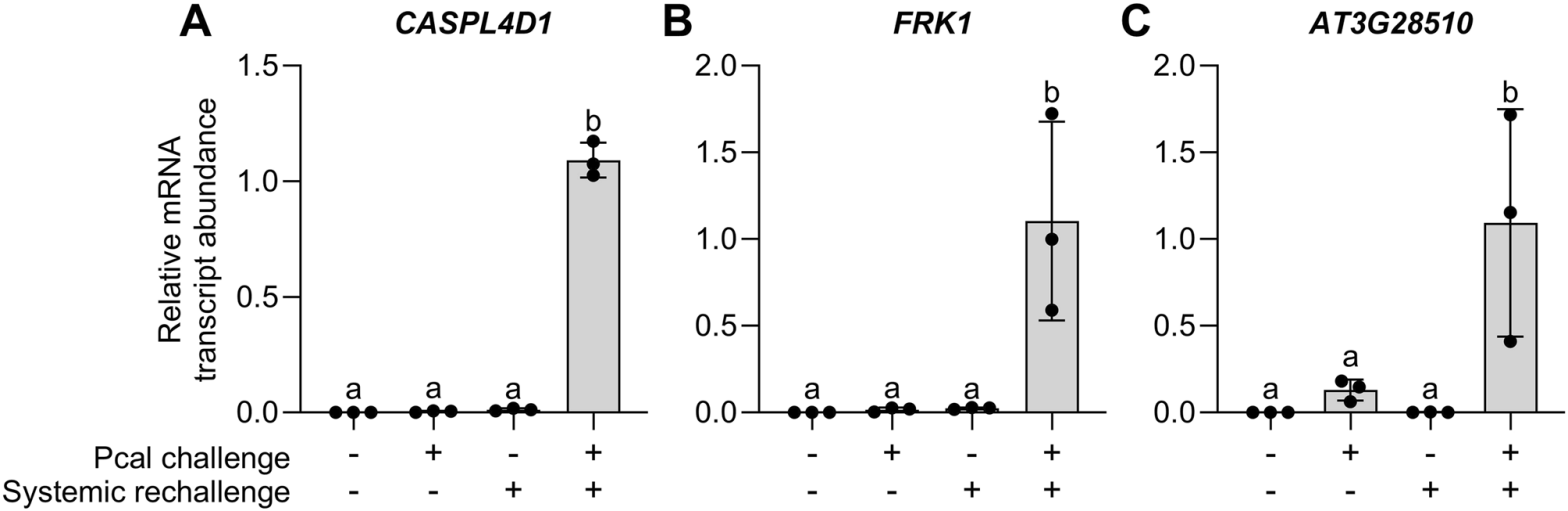
Readout genes of priming in Arabidopsis. *CASPL4D1* (**A**), *FRK1* (**B**), and *AT3G28510* (**C**) are particularly expressed in primed leaves when these have been rechallenged. Experimental setup, data and statistical analyses were done as in Fig. 2. (**A**), *P* < 0.0001; (**B**; **C**), *P* < 0.01.

Genes *CASPL4D1* (encoding Casparian strip domain-like protein 4D1; Fig. 4A), *FRK1* (a flg22-induced receptor-like kinase; Fig. 4B), and *AT3G28510* (P loop-containing nucleoside triphosphate hydrolases superfamily protein; Fig. 4C) show minimal to no expression in systemic leaves of control plants (condition 1), during priming (condition 2), or after rechallenge (condition 3). However, they exhibit strong expression in leaves rechallenged after priming (condition 4 in Fig. 1) (Fig. 4). Therefore, these genes can be classified as specific readout genes for priming. This classification also applies to *AT4G12500* (bifunctional inhibitor/lipid-transfer protein/seed storage 2S albumin superfamily protein), *WRKY6* (defense-related transcription factor), *WRKY53* (another defense-related transcription factor), *PME17* (pectin methyl esterase PME17), *WRKY29* (yet another defense-related transcription factor), *NRT2.6* (high-affinity nitrate transporter [NRT]2.6), and *RLP11* (receptor-like protein 11) (Supplementary Fig. S3A–G).

### Analyzing potential interactions among marker and readout proteins for priming

Revealing interaction networks of genes and proteins is instrumental in gaining a systems-level understanding of biological processes. To shed light on priming in Arabidopsis at this level, we utilized the Search Tool for the Retrieval of Interacting Genes/Proteins (STRING)^21^ available at https://string-db.org. Using this tool, we unveiled potential interaction networks of genes and proteins involved in priming in this plant.

**Figure 5:**
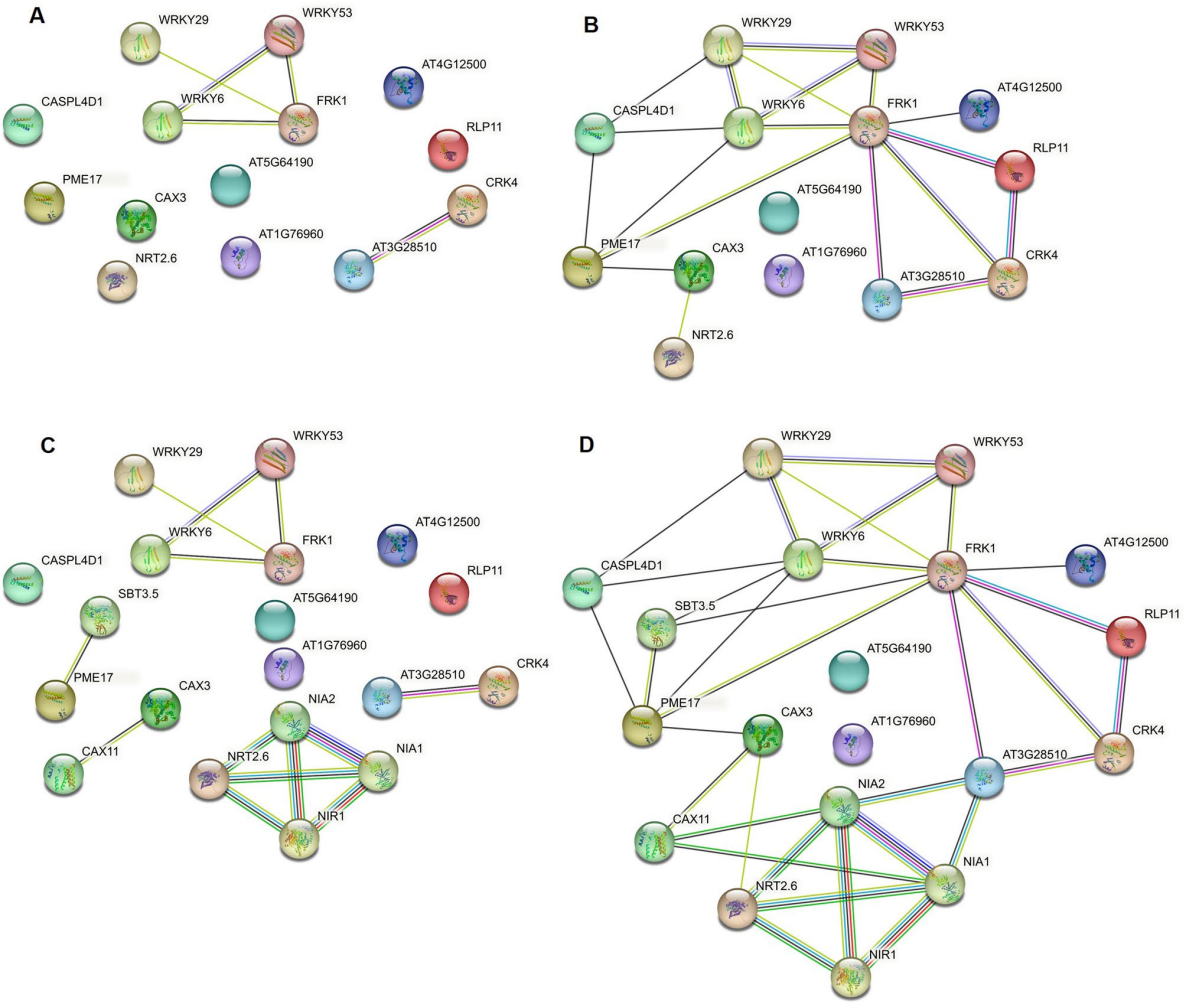
Associations of proteins encoded by marker or readout genes of priming. (**A**,** B**) Direct interactions. (**A**) Medium confidence (STRING value ≥ 0.4) or (**B**) low confidence (STRING value ≥ 0.15). (**C**, **D**) Interactions after allowing additional nodes. (**C**) Medium confidence and (**D**) low confidence. Nodes represent proteins. Unfilled nodes represent proteins with unknown 3D structure. Filled nodes represent proteins with known or predicted 3D structure. Edges represent protein–protein associations. Color code of known and predicted STRING interactions: cyan blue: from curated databases, purple: experimentally determined, green: gene proximity, red: gene fusions, blue: protein co-occurrence, yellow: text mining, black: co-expression, pale blue: protein homology. Figures were drawn using STRING database^21^ (https://string-db.org).

Our STRING analysis of validated specific marker genes (Fig. 2; Supplementary Fig. S1) and readout genes (Fig. 4; Supplementary Fig. S3) for priming predicted, with high confidence (STRING value ≥ 0.7), an equally-intense interaction network involving priming-readout proteins FRK1, WRKY6, and WRKY53 (Fig. 5A). With medium confidence (STRING value ≥ 0.4) we also observed potential interactions between FRK1 and WRKY29 (Fig. 5A). Of particular interest, we uncovered evidence of a strong interaction between priming-marker protein CRK4 and the P loop-containing nucleoside triphosphate hydrolases superfamily protein AT3G28510 (Fig. 5A). Notably, not only were the genes encoding these two proteins found to be co-expressed, but similar interactions were also observed between the orthologous genes in man, mouse and the eelworm *Caenorhabditis elegans*. These computational findings strongly suggest that CRK4 and the *AT3G28510*-encoded P loop-containing nucleoside triphosphate hydrolases superfamily protein may interact in Arabidopsis as well.

In addition to WRKY6, WRKY53, and WRKY29, FRK1 also appears to interact with moderate confidence with priming-readout proteins RLP11, the P loop-containing nucleoside triphosphate hydrolases superfamily protein encoded by priming-readout gene *AT3G28510*, and priming-marker protein CRK4 (Fig. 5B). FRK1 and WRKY6, with medium confidence, emerge as central nodes in an interaction network that encompasses all the priming-marker and readout proteins validated in this study, except for the neuronal PAS-domain protein encoded by *AT5G64190* and the protein with an unknown function encoded by *AT1G76960* (Fig. 5B).

When we expanded the network by lowering the stringency for node identification (Fig. 5C,D), our analysis, with moderate confidence, revealed a potential interaction involving the priming-marker protein CAX3 with CAX11, PME17 with subtilase-family protein SBT3.5 (Fig. 5C), and high-affinity nitrate transporter NRT2.6 (AT3G45060) with nitrate reductases NIA1 and NIA2, as well as nitrite reductase NIR1 (Fig. 5C). These four proteins, known for their roles in nitrogen metabolism^22^, are highly likely to interact. Based on gene neighborhood and co-expression, CAX11 also appears to interact with NIA1 and NIA2 with low confidence (Fig. 5D).

In summary, our STRING interaction network analysis suggests that the genes *WRKY53*, *WRKY6*, and *FRK1* often exhibit co-expression and close proximity (Fig. 5A–D). Notably, FRK1 appears to have a central role in the priming network of proteins. Indeed, many of the interactions predicted for the FRK1 protein in our STRING analysis have previously been experimentally demonstrated in pull-down assays or by solid phase array analysis (Table 2 and^23,24^).

**Table 2:** Experimentally demonstrated interactions of marker and readout proteins of priming (high confidence; STRING value ≥ 0.7).

Marker/readout protein	Experimentally confirmed interaction partners
FRK1 (AT2G19190)	AT1G17230
	BRL2 (AT2G01950)
	AT1G62950
	AT4G36180
	AT3G56370
	AT1G07560
	RHS16 (AT4G29180)
	NIK3 (AT1G60800)
	TMK1 (AT1G66150)
	AT2G01820
	SOBIR1 (AT2G31880)
	AT1G66830
	AT3G57830
	AT4G37250
	AT5G10020
	NPR1 (AT1G64280)
WRKY53 (AT4G23810)	TASTY (AT1G54040)
	MEKK1 (AT4G08500)
	WRKY30 (AT5G24110)
NRT2.6 (AT3G45060)	NRT3.1 (AT5G50200)
CAX3 (AT3G51860)	CAX1 (AT2G38170)
	T22C5.23 (AT1G27770)
	AT3G22910
	ACA10 (AT4G29900)
	ACA11 (AT3G57330)
CRK4 (AT3G45860)	AT5G46330
	ADF5 (AT2G16700)

Interestingly, for the neuronal PAS-domain protein AT5G64190, putative interaction partners were previously predicted with low confidence (0.15), based on *AT5G64190*’s co-expression with *AT4G19420* (encoding a pectin acetylesterase-family protein) and *AT3G03870* (encoding a protein with unknown function). Our disclosure of the protein interaction network was primarily based on the co-expression of encoding genes (Supplementary Dataset S1). Notably, co-expression and interactions have been rarely described or predicted for most of the marker and readout genes or proteins identified in this study. However, confirmation of gene co-expression and protein interaction is still pending.

### Predicted subcellular localization of marker and readout proteins for priming and their co-accumulating partners

To gain deeper insights into priming, our focus shifted towards analyzing the network of genes of greatest interest (Figs. 2, 3 and 4 and Supplementary Figs. S1–S3). We exported and further analyzed the results of our STRING analysis, considering an overall STRING score of ≥ 0.4, and searched for genes with the highest co-expression values (≥ 0.5) based on the co-expression of genes (using a threshold STRING co-expression score of 0.4). Subsequently, we utilized the Subcellular Localization database for Arabidopsis proteins (SUBA, version 5)^25^ available at http://suba.live/ to determine the localization of the proteins within plant cells (SUBA5, location consensus).

**Figure 6:**
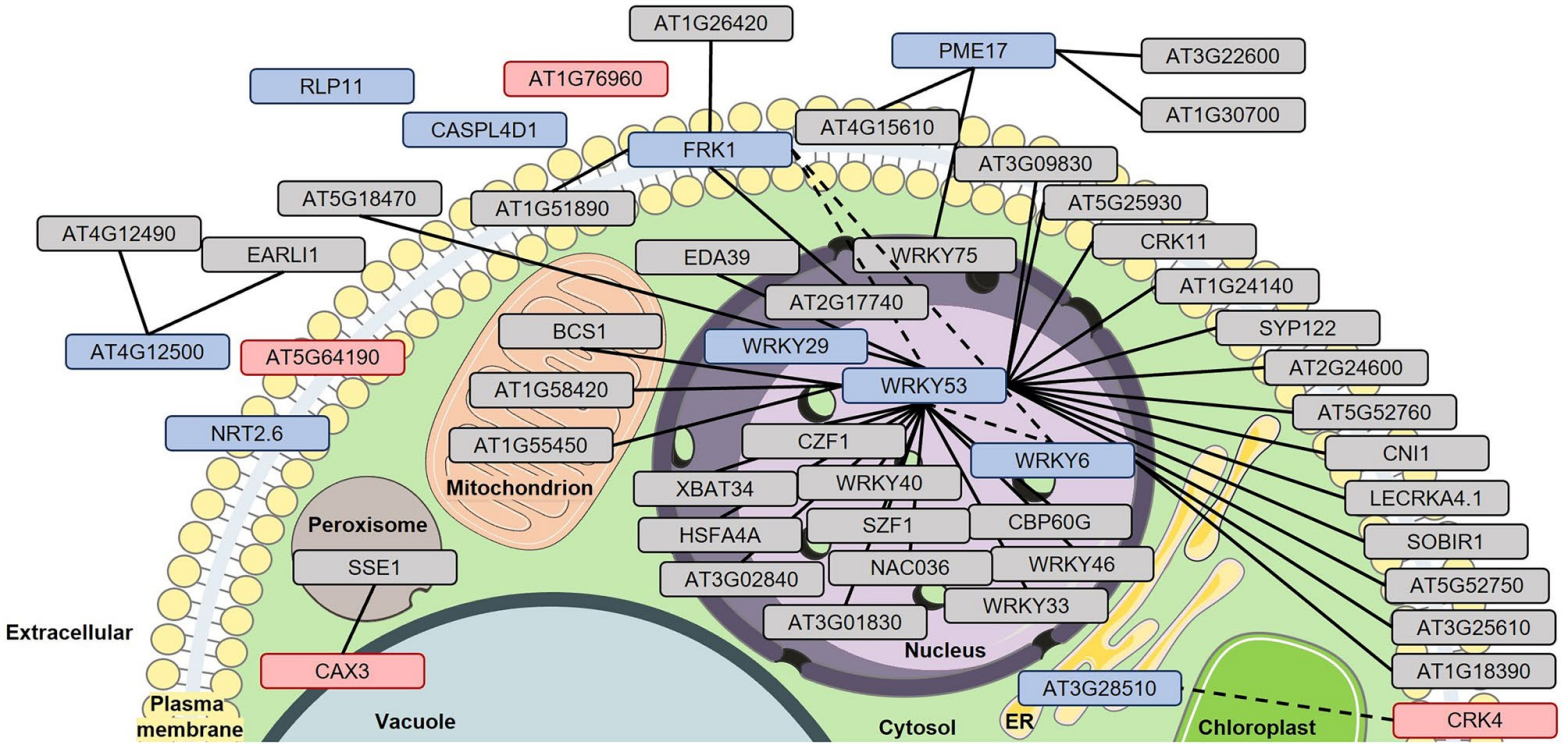
Subcellular localization of marker and readout proteins for priming and their supposed interaction partners. We used SUBA5 location consensus to determine the subcellular localization of marker and readout proteins for priming and their presumed-interacting protein partners. To keep clarity, we raised the STRING value to ≥ 0.5 when analyzing the co-expression with the in this work identified marker or readout genes for priming (Figs. 2, 3, 4; Supplementary Figs. S1–S3). In addition, only connections to the genes of interest (Figs. 2, 3, 4; Supplementary Figs. S1–S3) are indicated but not the connections between others. For more information, see Supplementary Dataset S1 (co-expression ≥ 500). ER, endoplasmic reticulum. Solid and dashed lines indicate co-expression of genes with a STRING value > 0.5 or < 0.5, respectively. Proteins in red boxes are marker proteins for priming, those in blue boxes are readout proteins for priming. Proteins in grey boxes are interaction partners of priming-marker or priming-readout proteins. Parts of the figure were drawn by using pictures from Servier Medical Art. Servier Medical Art by Servier is licensed under a Creative Commons Attribution 3.0 Unported License (https://creativecommons.org/licenses/by/3.0/).

We focused our analysis on candidates connected to the 14 genes depicted in Figs. 2, 4 and Supplementary Figures S1 and S3, applying a high cut-off (≥ 0.5 stringency). This approach allowed us to draw a subcellular priming map of proteins (Fig. 6) associated with the genes of our greatest interest (Figs. 2, 4; Supplementary Figs. S1 and S3). As shown in Fig. 6, most of the proteins that we presume to be connected, according to the STRING database, to the marker and readout proteins for priming are predicted to be localized to the plasma membrane (15 in total), nucleus (13), or extracellular space (6). Three of them are assigned to the mitochondria, whereas one each is predicted to be located in the cytosol and peroxisome (Fig. 6).

The predicted distribution of marker and readout proteins related to priming, along with their interacting proteins in at least five cellular compartments, further underscores the physiological complexity of priming. Furthermore, the predominance of these proteins in the plasma membrane and extracellular space substantiates their likely crucial role in plant defense within the apoplast/extracellular space.

### Mutation of priming-readout gene *NHL25* attenuates priming and SAR

**Figure 7:**
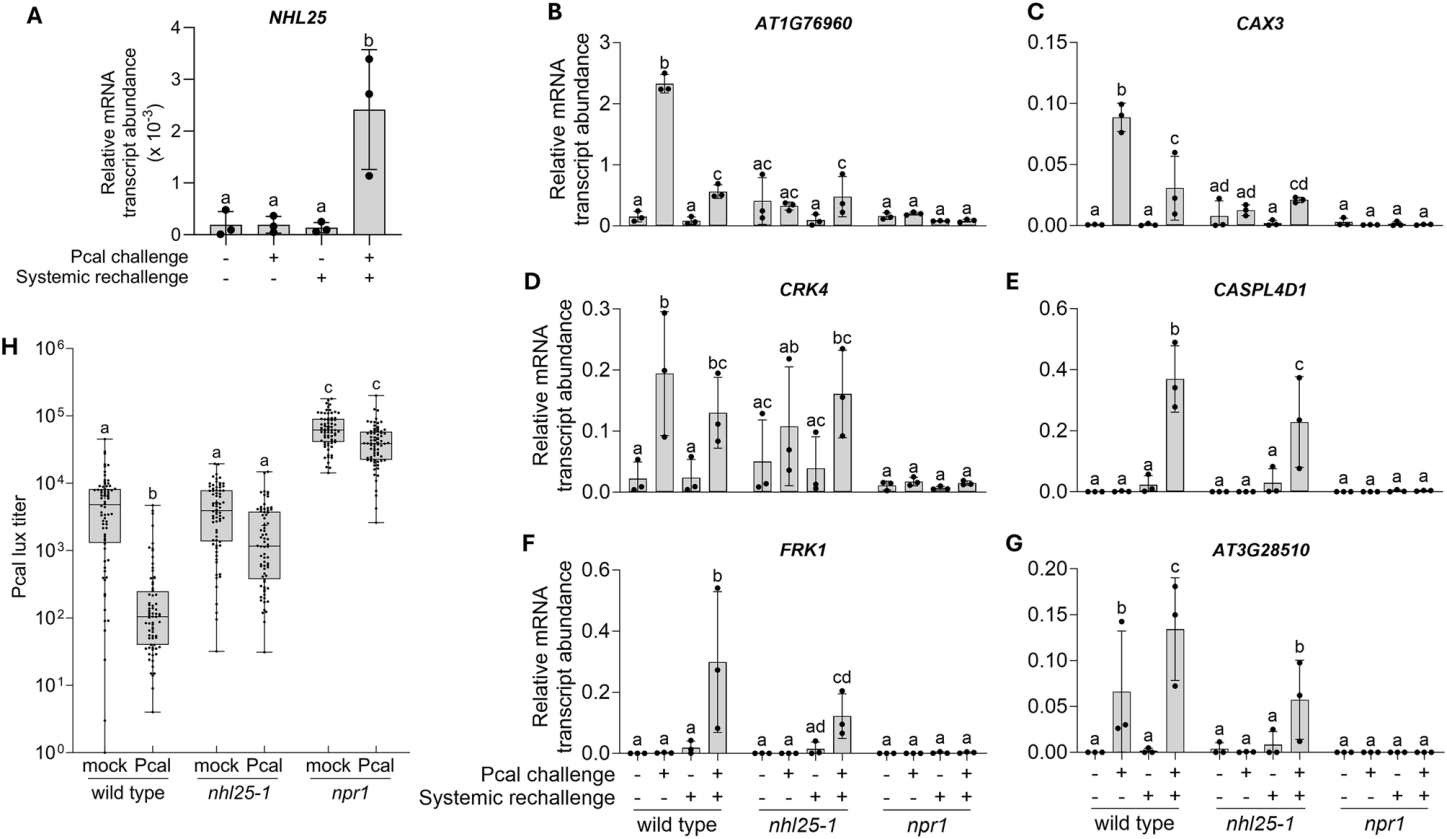
*NHL25* is a priming-readout gene whose mutation impairs priming and SAR. (**A**) *NHL25* is a readout gene for priming. Five-week-old wild-type plants were mock-inoculated (− Pcal) or infected with Pcal ( +) on three leaves. Three days later, distal leaves were left untreated (− systemic rechallenge) or infiltrated with water (+ systemic rechallenge). Three hours later, the systemic leaves were harvested and analyzed for the expression of the *NHL25* gene (normalized to *ACTIN2*). (**B**–**G**) Priming is attenuated or absent in the *nhl25-1* (SALK_113216) and *npr1* mutant. Five-week-old Arabidopsis plants were mock-inoculated (− Pcal) or infected with Pcal ( +) on three leaves. Three days later, systemic leaves were left untreated (− systemic rechallenge) or rechallenged by the infiltration of water (+ systemic rechallenge). Three hours later, untreated or infiltrated systemic leaves were harvested and analyzed for expression of the indicated marker (**B**–**D**) and readout (**E**–**G**) genes of priming. Relative mRNA transcript abundance was determined by RT-qPCR and normalized to *ACTIN2*. (**H**) SAR is absent in the *nhl25-1* and *npr1* mutant. Five-week-old plants were mock-inoculated or infected with Pcal on three leaves. Three days later, uninoculated systemic leaves were inoculated with Pcal lux. After another 3 days, the titer of Pcal lux was determined by measuring the luminescence in discs taken from systemic Pcal lux-inoculated leaves. For (**A**–**G**) mean values and SD of three independent experiments each with two plants are shown. For (**H**), data derived from three independent experiments each with eight biological replicates consisting of three leaves from an appropriately treated plant. Statistical significance was tested with Ordinary one-way ANOVA (**A**–**G**) or with Kruskal–Wallis test (**H**). **(A**; **E**), *P* < 0.01; (**B**; **C**; **D**; **F**; **G**), *P* < 0.05; (**H**), *P* < 0.001.

In Arabidopsis, the expression of *NHL25* is induced during incompatible interactions with pathogens^26^ and at least partly depends on salicylic acid^26^ which primes plants for enhanced defense^27,28^. Thus, *NHL25*, ranked #6 in the immunological condition 4 (primed and then rechallenged) data subset (named pC in^19^) (Table 1) could have a crucial role in priming and SAR. We considered this possibility and found that the *NHL25* gene exhibits minimal expression in untreated leaves, during priming, or after rechallenge (Fig. 7A). However, *NHL25* is expressed in primed leaves after they have been rechallenged (i.e., in condition 4) (Fig. 7A). Consequently, although it did not show up in our STRING and SUBA analyses at reasonable stringency, *NHL25* qualifies as a bona-fide readout gene for priming in Arabidopsis.

Surprisingly, although priming appears to be a complex physiological phenomenon (Fig. 6), the mutation of *NHL25* alone impaired the Pcal-induced systemic expression of priming-marker genes *AT1G76960* (encoding a protein with unknown function), *CAX3*, and *CRK4* (Fig. 7B–D), as well as of priming-readout genes *CASPL4D1*, *FRK1*, and *AT3G28510* (for a P loop-containing nucleoside triphosphate hydrolases superfamily protein) (Fig. 7E–G).

Moreover, mutation of *NHL25* impedes the development of SAR to Pcal in two independent *nhl25* and the priming/SAR-negative *npr1* mutant^29^ (Fig. 7H; Supplementary Fig. S4). Remarkably, unlike *npr1*, the two independent *nhl25* mutants do not exhibit enhanced basal susceptibility to Pcal (Fig. 7H; Supplementary Fig. S4).

Together the findings presented in Fig. 7B–H highlight *NHL25* as a previously unknown key gene in the primed SAR response of Arabidopsis.

## Discussion

Based on the patterns and levels of expression, we recommend the use of *AT1G76960*, *CAX3*, and *CRK4* as exclusive marker genes for priming in the Arabidopsis-Pcal interaction (Fig. 2). *PR1*, owing to its robust expression (Fig. 3A), is also a recommended marker gene for detecting priming in this plant. However, it’s important to note that *PR1* expression is not exclusive for the ‘only primed’ state.

For priming-readout genes (Fig. 4), we suggest utilizing *CASPL4D1*, *FRK1*, and *AT3G28510*. These genes provide valuable insight into priming. While the other marker (Fig. 3; Supplementary Figs. S1 and S2) and readout genes (Supplementary Fig. S3) identified in this study can also contribute to priming research in Arabidopsis, it’s worth noting that their overall expression levels are mostly lower than those of the genes recommended above.

CAX3 forms a complex with CAX1 (Fig. 5C), another Ca^2+^/H^+^ antiporter located in the tonoplast^30^. Both proteins have a vital role in maintaining Ca^2+^ homeostasis by facilitating the transport of Ca^2+^ ions from the cytosol into the vacuole^31^. Ca^2+^ serves as a second messenger^32^ and is a key activator of defense responses in plants^33^. In unstimulated cells, its concentration remains low, typically around 100 nM^32,33^. However, upon recognition of microbial patterns, there is a rapid influx of Ca^2+^ ions from the apoplast through specific Ca^2+^ channels^33^. This influx leads to an increase in cytosolic Ca^2+^ concentration, which is detected by Ca^2+^-binding proteins such as calmodulin, Ca^2+^-dependent protein kinases, and calcineurin B-like proteins. These proteins translate the Ca^2+^ signal into cellular responses that, e.g., help fight off infection^33^. However, high cytosolic Ca^2+^ concentrations can be toxic, and excess Ca^2+^ ions must be removed from the cytosol^32^. This process is facilitated by Ca^2+^-ATPases and Ca^2+^/H^+^ antiporters, including CAX1 and CAX3^34^. Therefore, the priming-linked expression of *CAX3* might serve as a preemptive response to anticipated challenges that could lead to threatening increases in cytosolic Ca^2+^ concentration.

Priming-marker protein CRK4 is located in the plasma membrane and associates with flg22 receptor FLS2. In a possible interaction with CRK6 and CRK36, it contributes to the priming for an enhanced flg22-induced oxidative burst and the defense against pathogenic Pseudomonads^35^. Similar to MPK3, MPK6, and FRK1, CRK4 during priming may accumulate in its inactive, yet activable form^12^. Upon perception of microbial patterns, such as flg22, more CRK4 molecules could be activated in primed cells compared to unprimed cells, potentially amplifying the transducing signal and leading to a more robust defense response^12^.

PR1 strongly accumulates after infection by various pathogens and upon treatment with certain chemicals, including salicylic acid^36,37^. In addition, PR1 has been used as a molecular marker for SAR in different plant species for a long time^38^. The PR1 protein binds to sterols and can cause cellular leakage^39^. By doing so it may exert antimicrobial activity that has been demonstrated both in vitro^40^ and in transgenic plants overexpressing *PR1*^41^. Arabidopsis PR1 proteins are secreted to the extracellular space^38^, where they could directly fight pathogens.

Priming-readout protein CASPL4D1^30^, a membrane protein that is localized to the chloroplast and plasma membrane, has been associated with Arabidopsis’ defense response to Pseudomonas before^42^. However, the exact mode of action and role of CASPL4D1 in plant immunity remain unclear. In contrast FRK1^30,43^ appears to be a central player in the priming network of proteins (Fig. 5; Table 2). Based on its expression patterns in the immunological conditions analyzed, FRK1 does not seem to accumulate during priming (Fig. 4B). Instead, *FRK1* expression is strongly activated when primed plants are rechallenged, indicating its role in a state of greatest distress.

It’s worth noting that *FRK1* has been identified as a reported target of transcription factor WRKY6^44^. Our STRING analysis further suggests that FRK1 likely interacts with WRKY53 and WRKY29 (Fig. 5A). *WRKY29* holds #9 in our top condition 4 list (primed and then rechallenged; pC, data subset in^19^) (Table 1), and *WRKY6* and *WRKY53* have reasonably high condition 4 (pC) and FAIRE values, along with relatively low cP (influence of priming on systemic rechallenge) values (Supplementary Table S1^19^), which categorizes them among the top ten priming-readout genes in our investigation (Fig. 4). *WRKY6*, *WRKY29*, and *WRKY53* belong to a large (> 70 members) family of loci encoding transcription factors with pivotal regulatory roles in plant immunity^13,45,46^. Priming of these three *WRKY* genes involves specific histone modifications in their promoter^13^ that create docking sites for chromatin-regulatory proteins, leading to local nucleosome eviction and the formation of nucleosome-free DNA (open chromatin). This open chromatin structure is a hallmark of primed gene promoters and plays a crucial role in the priming process^19,20,47^.

In the incompatible interaction of Arabidopsis with *P. syringae* expressing the bacterial effector gene *avrRpt2*, the expression of *AT3G28510* is dependent on the functional protein NDR1^48^ which is crucial for the resistance of Arabidopsis to bacterial and fungal pathogens^49^. The co-expression of NDR1-specific priming-readout genes *AT3G28510* and *FRK1* (Fig. 5B) suggests the involvement of the P loop-containing nucleoside triphosphate hydrolases superfamily protein AT3G28510 in Arabidopsis’ MAMP-response pathway. This further establishes *AT3G28510* as a robust priming-readout gene. However, it’s important to note that the role of the newly discovered interaction of AT3G28510 with priming-marker polypeptide CRK4 (Fig. 5A) requires experimental confirmation, as does the interaction between CAX3 and CAX11 (Fig. 5C,D), the interplay of FRK1 with RLP11 (Fig. 5B), and the interaction of the P loop-containing nucleoside triphosphate hydrolases superfamily protein AT3G28510 with CRK4 (Fig. 5C).

We find the suggested involvement of nitrate metabolism proteins NRT2.6, NIA1, NIA2, and NIR1 in priming intriguing (Fig. 5C). While NRT2.1, NRT2.2, NRT2.4, and NRT2.7 are established nitrate transporters whose genes are induced at low nitrogen levels, *NRT2.6* expression is primarily activated at high nitrogen levels^22^. Notably, a *nrt2.6* mutant did not exhibit a nitrate-related phenotype^22^ and even strong *NRT2.6* overexpression failed to rescue the nitrate-uptake defect of a *nrt2.1*–*nrt2.2* double mutant^22^. These findings suggest that Arabidopsis NRT2.6 may have other, or additional roles beyond nitrate transport. Interestingly, *NRT2.6* expression was induced in Arabidopsis upon infection with *Erwinia carotovora*, and plants with reduced *NRT2.6* expression displayed increased susceptibility to this pathogen^22^. The authors proposed a link between *NRT2.6* expression and Arabidopsis’ defense against *E. carotovora*, potentially through the accumulation of reactive oxygen species^22^. Our findings here support a role of *NRT2.6* in pathogen defense (Fig. 4I).

The apparent connection between nitrogen metabolism and priming may involve nitrate reductase-mediated release of nitric oxide, especially in conditions of excessive nitrate reductase activity^50,51^. During optimal nitrogen assimilation, cytoplasmic nitrate reductase reduces nitrate to nitrite. Nitrite is then transported to the chloroplast, where it is further reduced to NH_4_^+^ by nitrite reductase. Thus, NIA1 and NIA2 usually do not coincide with NIR1. However, the cytoplasmic nitrite concentration can increase significantly in the absence of an electrochemical gradient across the chloroplast envelope. This can occur when photosynthetic electron transport is impaired, often due to an attack by necrogenic pathogens.

We were particularly surprised to discover that a mutation in only *NHL25* impaired Pcal-induced priming and SAR in Arabidopsis (Fig. 7B–H). This finding suggests a critical role for NHL25 in both defense responses. Consistently, *NHL25* is not significantly induced during Arabidopsis interactions with compatible Pseudomonads (Fig. 7A)^26^. However, *NHL25* expression is robust when Arabidopsis interacts with *P*. *syringae* pv. tomato DC3000 carrying avirulence gene *avrRpm1*, *avrRpt2*, *avrB*, or *avrRps4*^26^. Furthermore, *NHL25* expression is activated upon rechallenging previously primed Arabidopsis plants with localized Pcal infection (Fig. 7A). Importantly, *NHL25* expression in Arabidopsis is only partly induced by salicylic acid^25^, and it appears that an additional rechallenge is required for full gene activation (Fig. 7A), which subsequently contributes to the fight against Pseudomonas infection (Fig. 7H).

## Methods

All methods were performed in accordance with relevant guidelines and regulations for plant specimens involved in the study in the manuscript.

### Cultivation of plants

Seeds of Arabidopsis (*A. thaliana*) wild-type and mutants *nhl25-1* (AT5G36970*;* SALK_113216) and *npr1-1* (AT1G64280), all in Col-0 genetic background, were obtained from the Nottingham Arabidopsis Stock Center (https://arabidopsis.info/). Plants were cultivated in soil and in short-day (8 h light, 120 µmol m^−2^ s^−1^) at 20 °C.

### Cultivation of bacteria

Pcal and *luxCDABE*-tagged Pcal (Pcal lux)^52^ were initially grown on King’s B agar medium (20 g L^−1^ tryptone, 10 mL L^−1^ glycerol, 1.5 g L^−1^ K_2_HPO_4_, and 1.5 g L^−1^ MgSO_4_)^53^ supplemented with 100 µg mL^−1^ streptomycin (Pcal) or 25 µg mL^−1^ kanamycin and 100 µg mL^−1^ rifampicin (Pcal lux) and 10 g L^−1^ agar. After incubation for 2 d at 28 °C, several colonies were selected and transferred to a 250-mL flask containing 50 mL King’s B liquid medium supplemented with the respective antibiotics. The flask was then incubated overnight at 28 °C with agitation at 220 rpm. The bacterial culture was subsequently pelleted by centrifugation at 1,800 g and 16 °C for 8 min, and the supernatant was removed. The pellet was resuspended in 50 mL of 10 mM MgCl_2_, and after another round of centrifugation, the pellet was once again resuspended in 50 mL of 10 mM MgCl_2_. A 1-mL portion of the bacterial suspension was diluted with 10 mM MgCl_2_ to an OD_600_ of 0.0002, resulting in a suspension of ~ 3 × 10^8^ colony-forming units (cfu) mL^−1^.

### Plant treatment

Plants were treated as described before^19,54^. Five-week-old Arabidopsis plants were treated using a syringe without a needle. Three leaves were infiltrated with either 10 mM MgCl_2_ (mock inoculation) or ~ 3 × 10^8^ cfu mL^−1^ Pcal in 10 mM MgCl_2_ (Pcal infection). For gene expression analysis, two systemic leaves per plant were either left untreated (no systemic rechallenge) or rechallenged by infiltrating tap water 72 h after the initial treatment (systemic rechallenge). Systemic leaves were harvested at 3 h after the systemic rechallenge and subjected to RT-qPCR analysis as described below.

### SAR assay

Using a syringe without a needle, three leaves of 5-week-old plants were infiltrated with either 10 mM MgCl_2_ (mock inoculation) or ~ 3 × 10^8^ cfu mL^−1^ Pcal in 10 mM MgCl_2_. After 72 h, three distal leaves of each plant received an additional infiltration with Pcal lux (~ 3 × 10^8^ cfu mL^−1^) in 10 mM MgCl_2_ as a systemic rechallenge. Subsequently, after another 72 h, leaf discs (0.5 cm diameter) were punched out from the inoculated systemic leaves and washed in 10 mM MgCl_2_. The luminescence of Pcal lux in the leaf discs was measured using CLARIOstar plate reader (BMG LABTECH, Ortenberg, Germany). In this assay, bacterial luminescence reflects bacterial multiplication^52,54^.

### Analysis of gene-specific mRNA transcript abundance by RT-qPCR

RNA was extracted from frozen leaves using the TRIZOL method^55^. Subsequently, 1 µg of RNA was treated with DNase (Thermo Fisher Scientific, Langerwehe, Germany) and subjected to cDNA synthesis using RevertAid reverse transcriptase (Thermo Fisher Scientific, Langerwehe, Germany). mRNA transcript abundance was quantified using RT-qPCR on a C1000 TouchTM Thermal Cycler (CFX 284TM Real-Time System, Bio-Rad, Feldkirchen, Germany) in 384-well Hard-Shell® PCR plates (Bio-Rad, Feldkirchen, Germany). Gene-specific primers (Supplementary Table S2) and iTaq™ SYBR® Green Supermix (Bio-Rad, Feldkirchen, Germany) were used for amplification. Data were normalized to the mRNA transcript level of *ACTIN2*.

### STRING database and SUBA5 analysis

The interaction between marker and readout proteins was examined using the STRING database^21^ (https://string-db.org) with varying levels of stringency, ranging from high to medium to low confidence (as described in the main text). Proteins with medium confidence scores in the general score category were further filtered based on their gene co-expression score (threshold > 0.5) and subsequently analyzed for their subcellular localization. A subset of proteins that exclusively interacted with the 14 verified marker and readout genes for priming was selected and their subcellular localization was determined using SUBA5^25^ (http://suba.live/). Subcellular descriptions were based on the SUBA location consensus SUBAcon.

### Statistical analysis

All experiments were conducted in triplicate or more. Statistical significance was assessed using GraphPad PRISM software (GraphPad Software, San Diego, CA, USA). Ordinary one-way ANOVA was employed for experiments with normal distribution, and the Kruskal–Wallis test was used for others. Statistical significance was considered when *P* < 0.05.

### Supplementary Information

Supplementary Information.

Supplementary Figures.

Supplementary Tables.

## Data Availability

The datasets analyzed during the current study are available in the European Nucleotide Archive repository (https://www.ebi.ac.uk/ena/browser/view), accession PRJEB32929.

## References

[1] Conrath U, Pieterse CMJ, Mauch-Mani B (2002). Priming in plant-pathogen interactions. Trends Plant Sci..

[2] Conrath U (2006). Priming: Getting ready for battle. Mol. Plant-Microbe Interact..

[3] Conrath U, Beckers GJM, Langenbach CJG, Jaskiewicz MR (2015). Priming for enhanced defense. Annu. Rev. Phytopathol..

[4] Ross AF (1961). Systemic acquired resistance induced by localized virus infections in plants. Virology.

[5] Ryals JA (1996). Systemic acquired resistance. Plant Cell.

[6] Van Hulten M, Pelser M, van Loon LC, Pieterse CMJ, Ton J (2006). Costs and benefits of priming for defense in Arabidopsis. Proc. Natl. Acad. Sci. U. S. A..

[7] Martinez-Medina A (2016). Recognizing plant defense priming. Trends Plant Sci..

[8] Beckers GJM, Conrath U (2007). Priming for stress resistance: From the lab to the field. Curr. Opin. Plant Biol..

[9] Tateda C (2014). Salicylic acid regulates Arabidopsis microbial pattern receptor kinase levels and signaling. Plant Cell.

[10] Asai T (2002). MAP kinase signaling cascade in Arabidopsis innate immunity. Nature.

[11] Bender KW, Zipfel C (2023). Paradigms of receptor kinase signaling in plants. Biochem. J..

[12] Beckers GJM (2009). Mitogen-activated protein kinases 3 and 6 are required for full priming of stress responses in *Arabidopsis thaliana*. Plant Cell.

[13] Jaskiewicz M, Conrath U, Peterhänsel C (2011). Chromatin modification acts as a memory for systemic acquired resistance in the plant stress response. EMBO Rep..

[14] Luna E, Bruce TJA, Roberts MR, Flors V, Ton J (2012). Next-generation systemic acquired resistance. Plant Physiol..

[15] Conrath U (2011). Molecular aspects of defense priming. Trends Plant Sci..

[16] Katagiri F, Thilmony R, He SY (2002). The Arabidopsis thaliana-Pseudomonas syringae interaction.

[17] Baltrus DA (2011). Dynamic evolution of pathogenicity revealed by sequencing and comparative genomics of 19 *Pseudomonas syringae* isolates. PLoS Pathog..

[18] Sarris PF (2013). Comparative genomics of multiple strains of Pseudomonas cannabina pv alisalensis, a potential model pathogen of both monocots and dicots. PLoS ONE.

[19] Baum S (2019). Isolation of open chromatin identifies regulators of systemic acquired resistance. Plant Physiol..

[20] Baum S, Reimer-Michalski E-M, Jaskiewicz MR, Conrath U (2020). Formaldehyde-assisted isolation of regulatory DNA elements from Arabidopsis leaves. Nat. Protoc..

[21] Szklarczyk D (2021). The STRING database in 2021: customizable protein-protein networks, and functional characterization of user-uploaded gene/measurement sets. Nucleic Acids Res..

[22] Dechorgnat J, Patrit O, Krapp A, Fagard M, Daniel-Vedele F (2012). Characterization of the *NRT2.6* gene in *Arabidopsis thaliana*: A link with plant response to biotic and abiotic stress. PLsS ONE.

[23] Smakowska-Luzan E (2018). An extracellular network of Arabidopsis leucine-rich repeat receptor kinases. Nature.

[24] Mott GA (2019). Map of physical interactions between extracellular domains of Arabidopsis leucine-rich repeat receptor kinases. Sci. Data.

[25] Hooper C (2022). Subcellular localization database for Arabidopsis proteins version 5.

[26] Varet A (2002). NHL25 and NHL3, two NDR1/HIN1-1ike genes in *Arabidopsis thaliana* with potential role(s) in plant defense. Molec. Plant-Microbe Interact..

[27] Thulke O, Conrath U (1998). Salicylic acid has a dual role in the activation of defense-related genes in parsley. Plant J..

[28] Katz V, Fuchs A, Conrath U (2002). Pretreatment with salicylic acid primes parsley cells for enhanced ion transport following elicitation. FEBS Lett..

[29] Kohler A, Schwindling S, Conrath U (2002). Benzothiadiazole-induced priming for potentiated responses to pathogen infection, wounding, and infiltration of water into leaves requires the *NPR1/NIM1* gene in Arabidopsis. Plant Physiol..

[30] Berardini TZ (2015). The Arabidopsis information resource: Making and mining the “gold standard” annotated reference plant genome. Genesis.

[31] Cho D (2012). Vacuolar CAX1 and CAX3 influence auxin transport in guard cells via regulation of apoplastic pH. Plant Physiol..

[32] White PJ, Broadley MR (2003). Calcium in plants. Ann. Bot..

[33] Zhang L, Du L, Poovaiah BW (2014). Calcium signaling and biotic defense responses in plants. Plant Signal. Behav..

[34] Hirschi K (2001). Vacuolar H^+^/Ca^2+^ transport: Who’s directing the traffic?. Trends Plant Sci..

[35] Yeh YH, Chang YH, Huang PY, Huang JB, Zimmerli L (2015). Enhanced Arabidopsis pattern-triggered immunity by overexpression of cysteine-rich receptor-like kinases. Front. Plant Sci..

[36] Ward ER (1991). Coordinate gene activity in response to agents that induce systemic acquired resistance. Plant Cell.

[37] Conrath U, Chen Z, Ricigliano JR, Klessig DF (1995). Two inducers of plant defense responses, 2,6-dichloroisonicotinic acid and salicylic acid, inhibit catalase activity in tobacco. Proc. Natl. Acad. Sci. U. S. A..

[38] Van Loon LC, Rep M, Pieterse CJM (2006). Significance of inducible defense-related proteins in infected plants. Annu. Rev. Phytopathol..

[39] Gamir J (2017). The sterol-binding activity of pathogenesis-related protein 1 reveals the mode of action of an antimicrobial protein. Plant J..

[40] Niderman T (1995). Pathogenesis-related PR-1 proteins are antifungal. Isolation and characterization of three 14-kilodalton proteins of tomato and of a basic PR-1 of tobacco with inhibitory activity against *Phytophthora infestans*. Plant Physiol..

[41] Alexander D (1993). Increased tolerance to two oomycete pathogens in transgenic tobacco expressing pathogenesis-related protein 1a. Proc. Natl. Acad. Sci. U. S. A..

[42] Mohr PG, Cahill DM (2007). Suppression by ABA of salicylic acid and lignin accumulation and the expression of multiple genes, in Arabidopsis infected with *Pseudomonas syringae* pv. tomato. Func. Integr. Genomics.

[43] Po-Wen C, Singh P, Zimmerli L (2013). Priming of the Arabidopsis pattern-triggered immunity response upon infection by necrotrophic *Pectobacterium carotovorum* bacteria. Molec. Plant Pathol..

[44] Robatzek S, Somssich IE (2002). Targets of AtWRKY6 regulation during plant senescence and pathogen defense. Genes Dev..

[45] Wang D, Amornsiripanitch N, Dong X (2006). A genomic approach to identify regulatory nodes in the transcriptional network of systemic acquired resistance in plants. PLOS Pathog..

[46] Tsuda K, Somssich IE (2015). Transcriptional networks in plant immunity. New Phytol..

[47] Giresi PG, Lieb JD (2009). Isolation of active regulatory elements from eukaryotic chromatin using FAIRE (Formaldehyde Assisted Isolation of Regulatory Elements). Methods.

[48] Sato M (2007). A high-performance, small-scale microarray for expression profiling of many samples in Arabidopsis-pathogen studies. Plant J..

[49] Century KS, Holub EB, Staskawicz BJ (1995). NDR1, a locus of *Arabidopsis thaliana* that is required for disease resistance to both a bacterial and a fungal pathogen. Proc. Natl. Acad. Sci. USA.

[50] Desikan R, Griffiths R, Hancock J, Neill S (2002). A new role for an old enzyme: Nitrate reductase-mediated nitric oxide generation is required for abscisic acid-induced stomatal closure in *Arabidopsis thaliana*. Proc. Natl. Acad. Sci. U. S. A..

[51] Yamamoto-Katou A, Katou S, Yoshioka H, Doke N, Kawakita K (2006). Nitrate reductase is responsible for elicitin-induced nitric oxide production in *Nicotiana benthamiana*. Plant Cell Physiol..

[52] Fan J, Crooks C, Lamb C (2008). High-throughput quantitative luminescence assay of the growth in planta of *Pseudomonas syringae* chromosomally tagged with *Photorhabdus luminescens luxCDABE*. Plant J..

[53] King EO, Ward MK, Raney DE (1954). Two simple media for the demonstration or pyrocyanin and fluorescin. J. Lab. Clin. Med..

[54] Gruner K, Zeier T, Aretz C, Zeier J (2018). A critical role for Arabidopsis MILDEW RESISTANCE LOCUS O2 in systemic acquired resistance. Plant J..

[55] Chomczynski P (1993). A reagent for the single-step simultaneous isolation of RNA, DNA and proteins from cell and tissue samples. BioTechniques.

