# Supplementary material for: Marker and readout genes for defense priming in Pseudomonas cannabina pv. alisalensis interaction aid understanding systemic immunity in Arabidopsis
Source: Sci Rep. 2024 Feb 12;14:3489. doi: 10.1038/s41598-024-53982-5 (PMC10861594; doi:10.1038/s41598-024-53982-5)
Supplement: Supplementary file 2 — Supplementary Figures. [file 41598_2024_53982_MOESM2_ESM.pptx]

## Slide 1
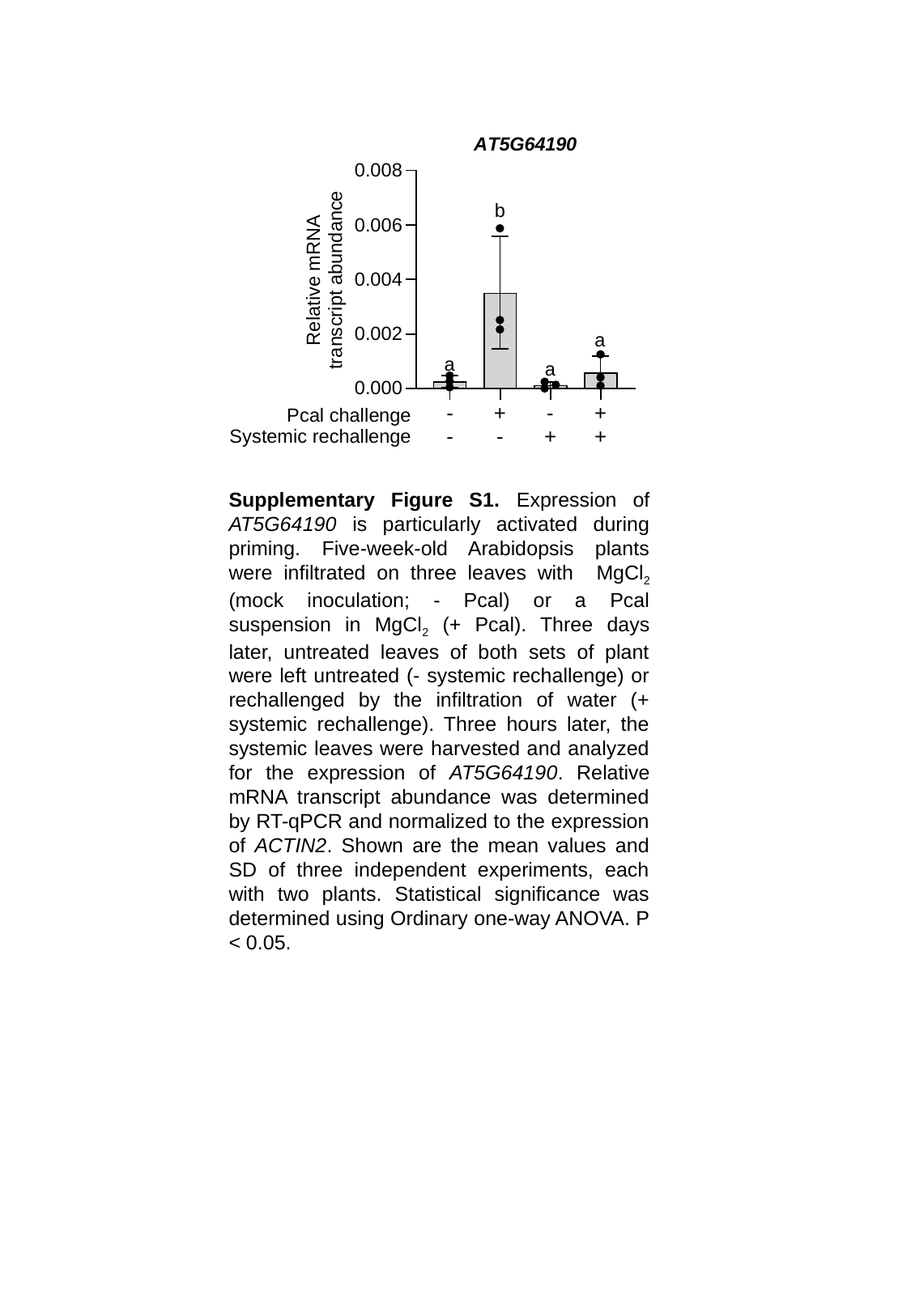

Supplementary Figure S1. Expression of AT5G64190 is particularly activated during priming. Five-week-old Arabidopsis plants were infiltrated on three leaves with MgCl2 (mock inoculation; - Pcal) or a Pcal suspension in MgCl2 (+ Pcal). Three days later, untreated leaves of both sets of plant were left untreated (- systemic rechallenge) or rechallenged by the infiltration of water (+ systemic rechallenge). Three hours later, the systemic leaves were harvested and analyzed for the expression of AT5G64190. Relative mRNA transcript abundance was determined by RT-qPCR and normalized to the expression of ACTIN2. Shown are the mean values and SD of three independent experiments, each with two plants. Statistical significance was determined using Ordinary one-way ANOVA. P < 0.05.

## Slide 2
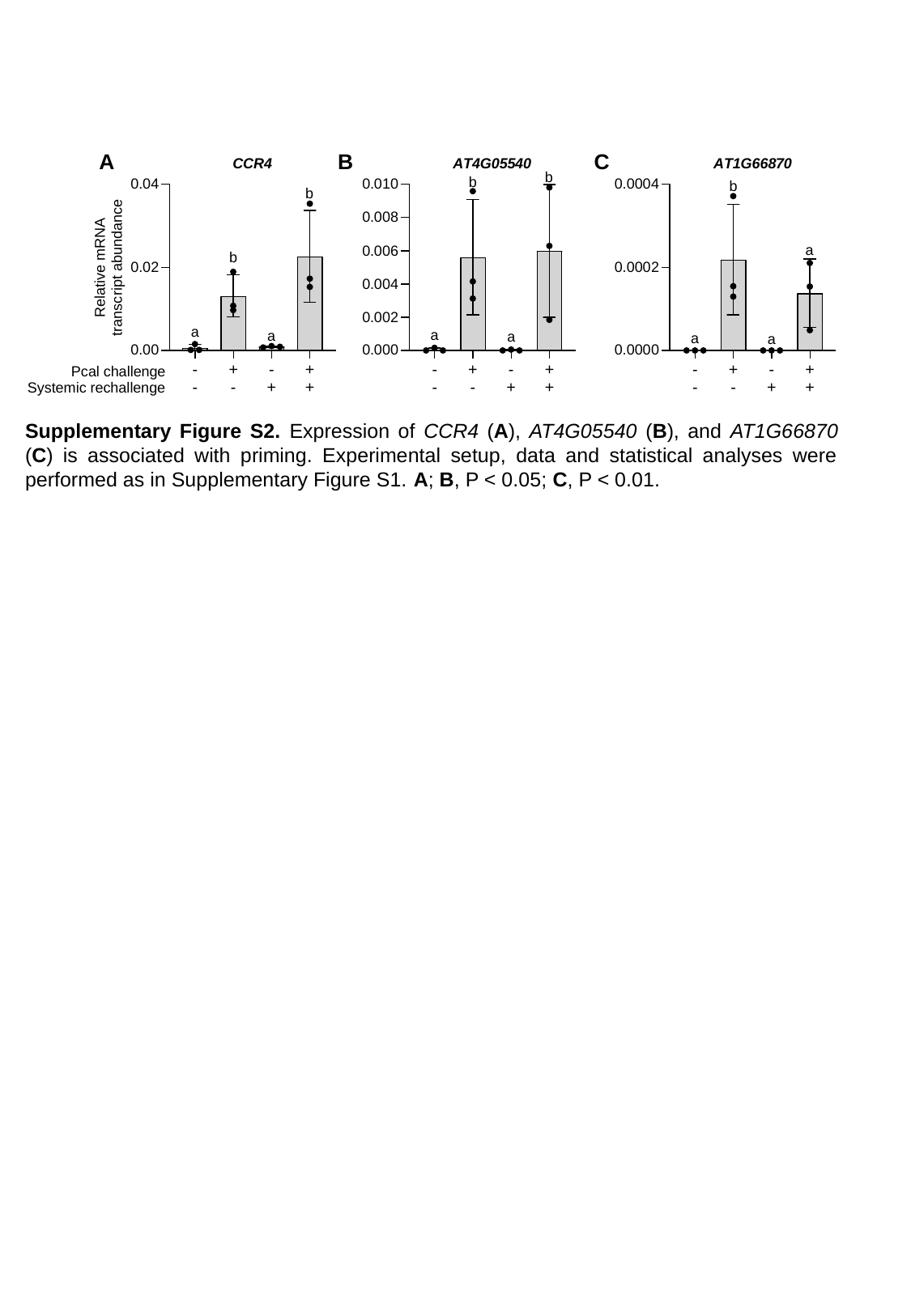

Supplementary Figure S2. Expression of CCR4 (A), AT4G05540 (B), and AT1G66870 (C) is associated with priming. Experimental setup, data and statistical analyses were performed as in Supplementary Figure S1. A; B, P < 0.05; C, P < 0.01.

## Slide 3
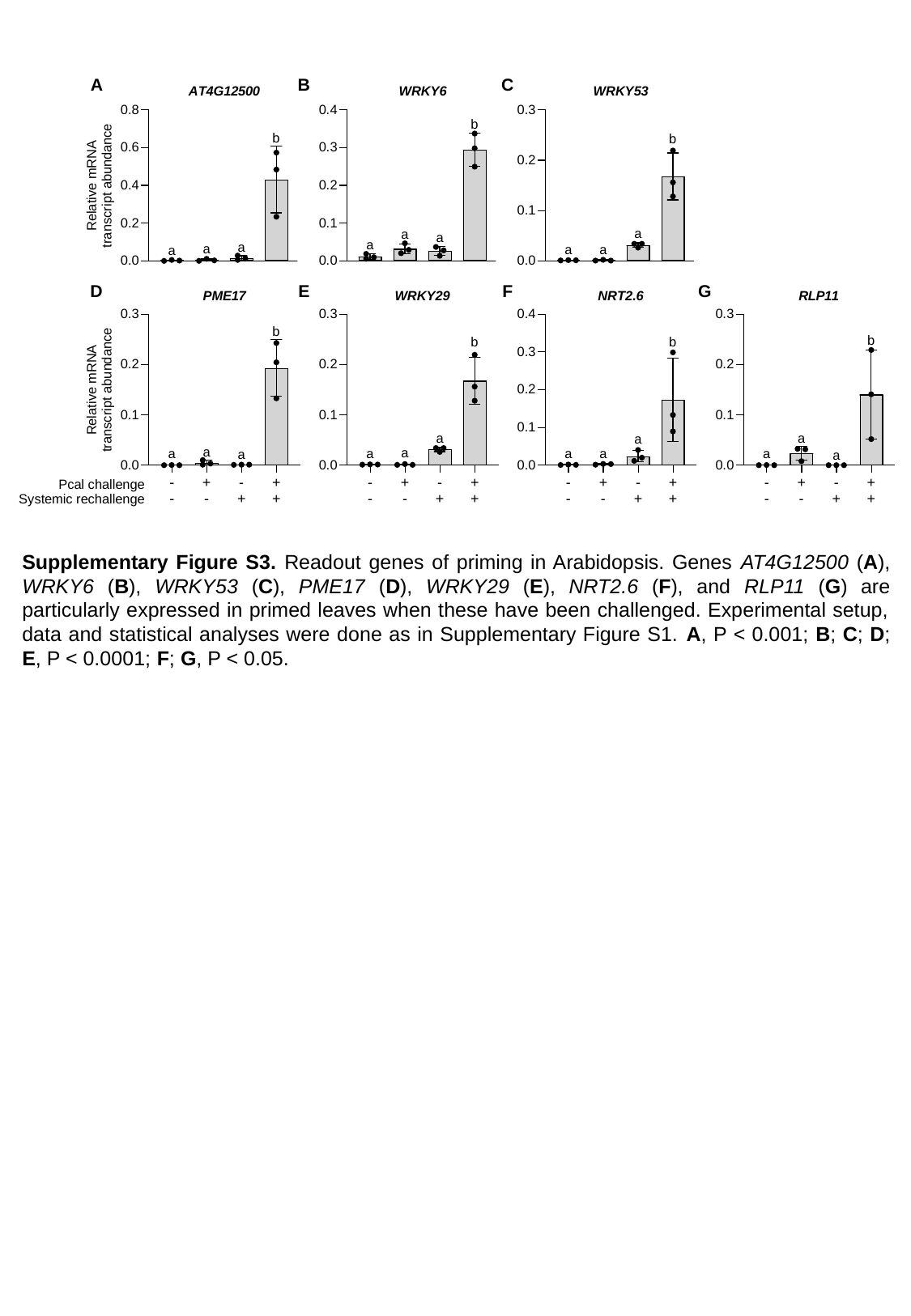

Supplementary Figure S3. Readout genes of priming in Arabidopsis. Genes AT4G12500 (A), WRKY6 (B), WRKY53 (C), PME17 (D), WRKY29 (E), NRT2.6 (F), and RLP11 (G) are particularly expressed in primed leaves when these have been challenged. Experimental setup, data and statistical analyses were done as in Supplementary Figure S1. A, P < 0.001; B; C; D; E, P < 0.0001; F; G, P < 0.05.

## Slide 4
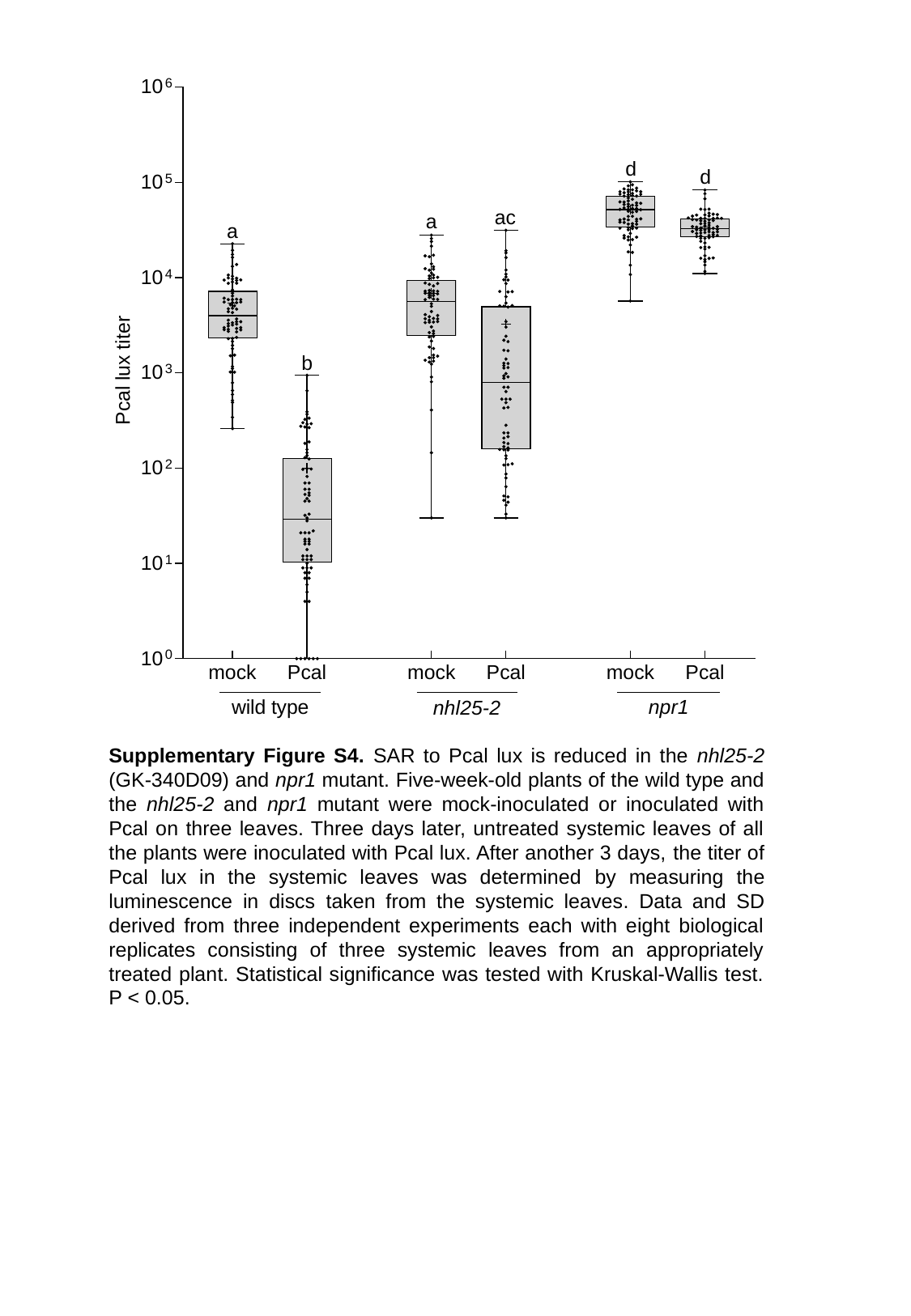

Supplementary Figure S4. SAR to Pcal lux is reduced in the nhl25-2 (GK-340D09) and npr1 mutant. Five-week-old plants of the wild type and the nhl25-2 and npr1 mutant were mock-inoculated or inoculated with Pcal on three leaves. Three days later, untreated systemic leaves of all the plants were inoculated with Pcal lux. After another 3 days, the titer of Pcal lux in the systemic leaves was determined by measuring the luminescence in discs taken from the systemic leaves. Data and SD derived from three independent experiments each with eight biological replicates consisting of three systemic leaves from an appropriately treated plant. Statistical significance was tested with Kruskal-Wallis test. P < 0.05.
