# Supplementary material for: Marker and readout genes for defense priming in Pseudomonas cannabina pv. alisalensis interaction aid understanding systemic immunity in Arabidopsis
Source: Sci Rep. 2024 Feb 12;14:3489. doi: 10.1038/s41598-024-53982-5 (PMC10861594; doi:10.1038/s41598-024-53982-5)
Supplement: Supplementary file 3 — Supplementary Tables. [file 41598_2024_53982_MOESM3_ESM.docx]

**Supplementary Table S1.** ncP, pC, FAIRE, and cP values of priming-readout genes *WRKY6* and *WRKY53*.

| **Gene locus** | **Gene symbol** | **Gene description** | **ncP logFC** | **pC logFC** | **cP logFC** | **FAIRE logRatio** |
| --- | --- | --- | --- | --- | --- | --- |
| *AT1G62300* | *WRKY6* | Encodes transcription factor WRKY6. Regulates *PHOSPHATE1* expression in response to mild phosphate stress. | 0.86 | 4.04 | 1.46 | 3.16 |
| *AT4G23810* | *WRKY53* | Encodes transcription factor WRKY53; WRKY53 and  CRK5 are antagonistic regulators of chlorophyll synthesis/degradation, senescence, and stomatal conductance. | 2.40 | 2.29 | 1.38 | 2.84 |

ncP, genes expressed during priming; pC, genes expressed after challenge of primed leaves; cP, genes expressed because of priming in systemic challenge condition. logFC, logFold change; FAIRE, formaldehyde-assisted isolation of regulatory DNA elements. Table based on Supplemental Dataset S1 of Baum et al. [19], gene symbols and descriptions were updated according to TAIR.

**Supplementary Table S2.** Gene-specific DNA primer sequences used in RT-qPCR analyses.

| **Data set** | **Gene locus** | **Gene symbol** | **Gene-specific DNA primer sequence 5´- 3´** | |
| --- | --- | --- | --- | --- |
|  |  |  | **Forward** | **Reverse** |
| Top exclusive marker genes for priming | *AT3G25010* | *RLP41* | GGAAATGCAGGGCTTTGTGG | CATACCCTATTGCCACGCCT |
|  | *AT5G64190* |  | CATGACAATGACCCCGTGGA | AACCTCTCTTCCTCCAACGC |
|  | *AT2G32680* | *RLP23* | CAGGAAGCTTGCCACCAAAC | TAACCATAGCCGCCTTCGTC |
|  | *AT5G47850* | *CCR4* | GATATCGCGGCTGGATTCCA | AGCTCAGGACCGTGGTAGAT |
|  | *AT3G45860* | *CRK4* | GCCTCGCTCTACTCCTCAAC | CAGAGGAGTTCCGGCCTTTT |
|  | *AT1G76960* |  | GGCGCTTCTCATAATCTCCCA | GGGTAACTACGCGGATACGG |
| Top priming-responsive genes | *AT2G14610* | *PR1* | GGTAGCGGTGACTTGTCTGG | CAAACTCCATTGCACGTGTT |
|  | *AT1G71390* | *RLP11* | CCTTCGCTTCACTTTTGCCG | TGACACCGTCCCAAGAACAG |
| Top genes expressed because of priming in systemic rechallenge condition | *AT4G05540* |  | GGGGATAGACGAAAGGGGGA | GACTGCCTCCAAACTCCGTT |
|  | *AT2G14610* | *PR1* | GGTAGCGGTGACTTGTCTGG | CAAACTCCATTGCACGTGTT |
|  | *AT1G66870* |  | TGTTTCCGCAATTGACAGTTGTA | TTGGCCGAGACATGAAGAGG |
|  | *AT3G51860* | *CAX3* | CTTGGTTCTTGGCACTTCCC | CAAATGACACAACAGGCCCA |
|  | *AT3G28510* |  | AGCTAACGACGGTGAAGGAT | TTTTCTTCTTTCGCTGCCCC |
| Top genes expressed after rechallenge in primed leaves | *AT3G45060* | *NRT2.6* | ATCGGAAATGCTGGAGTTGC | AGCATAAGTGAGAAGGCGGT |
|  | *AT2G39530* | *CASPL4D1* | AATCTCCCAATTTGCCACCG | TTGCTCACTCCAAATCCTGC |
|  | *AT4G12500* |  | TTGTCTCTGCACTGCCCTTA | TGGAAATCAGACGGAAGCCT |
|  | *AT5G36970* | *NHL25* | ACAGGAAGTATACCGTTGCGG | CACACTGTTAGCAGCCAAGC |
|  | *AT2G45220* | *PME17* | CCGGAAGCAAAAGTGTTGGA | CTACCGCTTGTTCGTTGCTT |
|  | *AT2G19190* | *FRK1* | TGGCTTTGTTTAATATTCAGATG | GTTTTCTGGGAAAATTGTATGC |
|  | *AT4G23550* | *WRKY29* | TTTCACCTTCGTTTTGCCTACC | CGAGCTCATCTAAGCCACTTGTC |
| Other | *AT3G18780* | *ACTIN2* | GGTAACATTGTGCTCAGTGGTGG | GGTGCAACGACCTTAATCTTCAT |
|  | *AT1G62300* | *WRKY6* | ACTTCACGGTCATTATCTCCAGC | TGAATTTAGGTTTCCGGTGAGTC |
|  | *AT4G23810* | *WRKY53* | CTCCATCGGCAAACTCTTCAC | CCGAGCGTACAACTTATTCCG |
